# Supplementary material for: Inverse resource allocation between vision and olfaction across the genus Drosophila
Source: Nat Commun. 2019 Mar 11;10:1162. doi: 10.1038/s41467-019-09087-z (PMC6411718; doi:10.1038/s41467-019-09087-z)
Supplement: Supplementary file 1 — Supplementary Information [file 41467_2019_9087_MOESM1_ESM.pdf]

Supplementary Information

Inverse resource allocation between vision and olfaction across the genus *Drosophila*

Keesey et al.

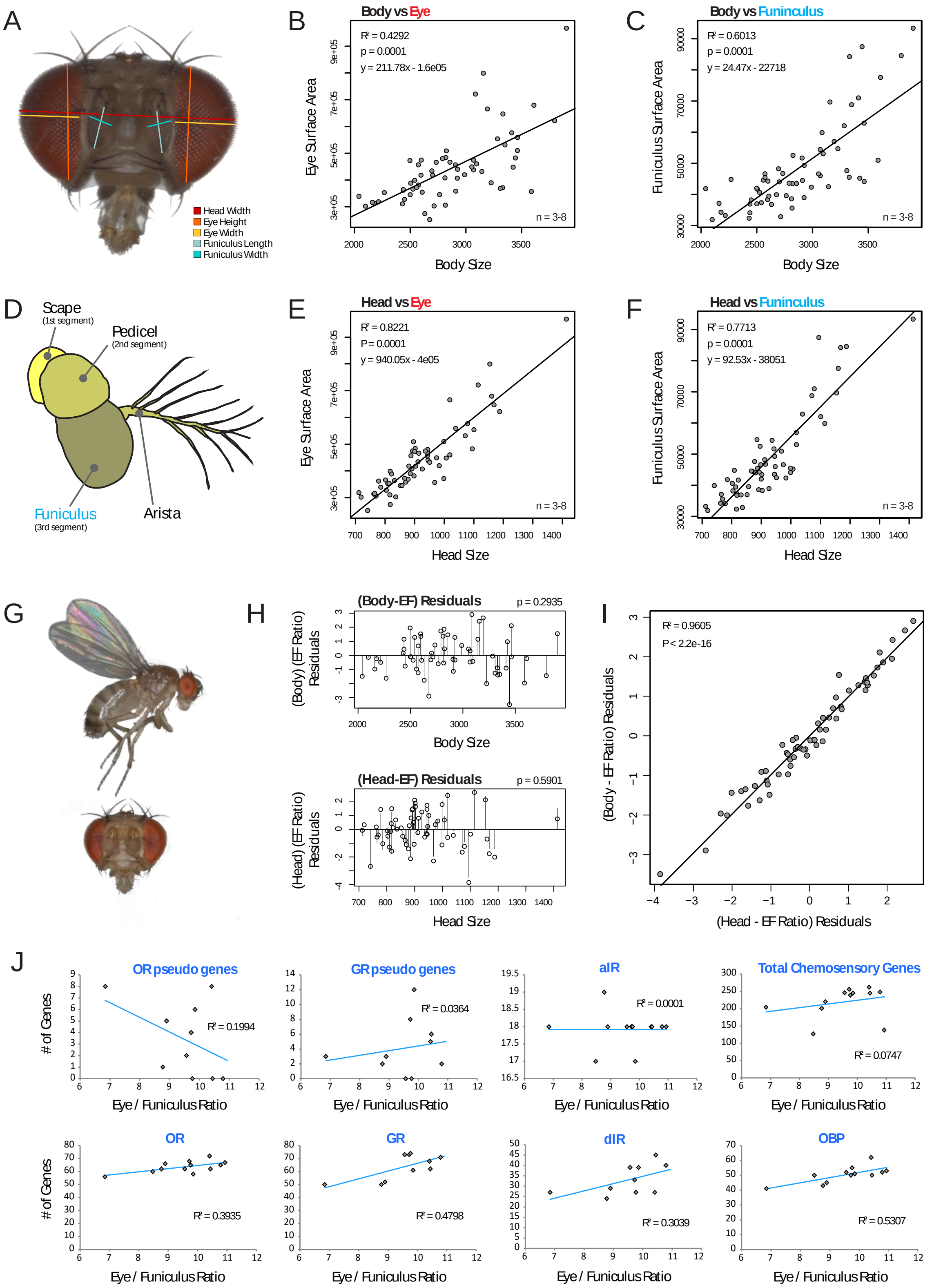

Supplementary Figure 1: External morphometrics from 62 species and functional chemoreceptor genes. (A) Example of measurements taken to calculate eye and funiculus surface area for each species. (B,C) Eye and funiculus surface area (μm^2) as compared to body size for each species. (D) Diagram of the *Drosophila* antenna, highlighting the 3rd antennal segment, also known as the funiculus (where the majority of chemosensory sensilla are located). (E,F) Eye and funiculus

surface area (μm^2) as compared to head size for each species. (G) Example of lateral and frontal views (*Drosophila melanogaster*), which were used to measure the body, head, eye and funiculus. (H) Plotting of the residuals, where neither body nor head size significantly correlate with the EF ratio trait, suggesting that this trait does not simply scale allometrically with respect to body and head size. (I) Residuals of head and body have highly similar deviations from EF-ratio, supporting that body and head size are highly correlated across all species. (J) Different chemosensory genes from 12-14 *Drosophila* species genomes and their correlation to the EF ratio [1], where number of olfactory pseudogenes, for example, does not suggest a sensory tradeoff. (Data are provided at doi.org/10.17617/3.1D).

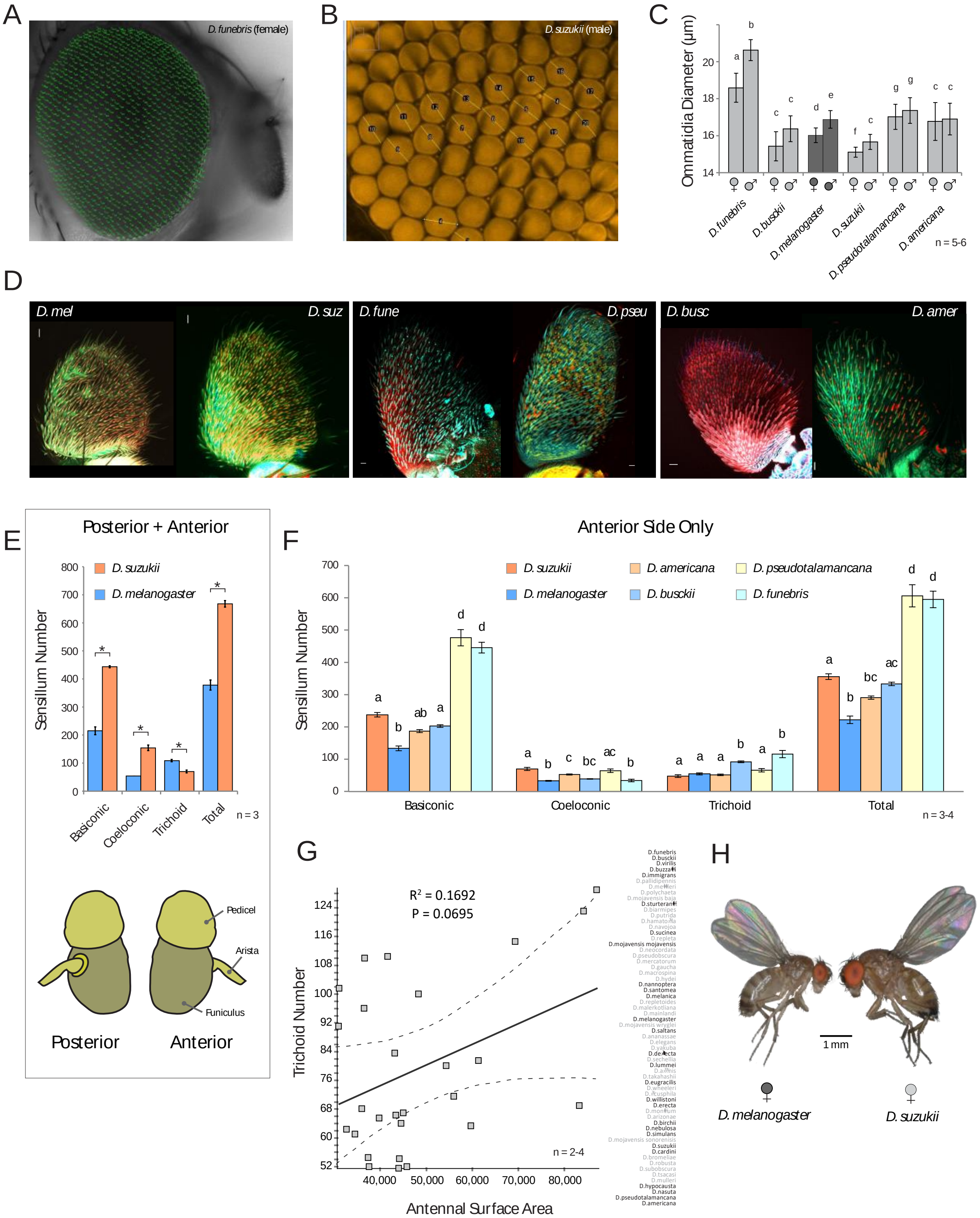

Supplementary Figure 2: Visual and olfactory sensory receptor measurements. (A) Example of ommatidium counts from photomontage of lateral view of *D. funebris* female head. (B) Examples of measurements taken to compare ommatidium diameters between species. (C) Ommatidia diameters. Means with the same letter are not significantly different from each other (ANOVA with Tukey-Kramer multiple comparison test). Error bars represent standard deviation. (D) Shown are examples of the images used for sensillum counts that were taken from stacked lambda mode scans (maximum intensity projections) of the anterior portion of the antenna for all 6 species examined. (E) Absolute sensillum counts from both sides of the antenna, as well as a diagram of anterior and posterior sides. Red to yellow color

signifies vision or visual bias, while blue indicates olfaction or olfactory species. An asterisk denotes statistical significance between two groups (*P ≤ 0.05, ***P ≤ 0.001; T-test). (F) Sensillum counts from lambda scans from only the anterior side of the antenna and the comparisons between all six species. Means with the same letter are not significantly different from each other (ANOVA with Tukey-Kramer multiple comparison test). Error bars represent standard deviation. (G) There is no correlation between trichoid number and antennal surface area, arguing against the idea that larger species necessarily have more trichoids. (H) Absolute size comparisons between two species, illustrating the differences in body, head, and eye morphology, where the body of the *D. suzukii* female is 1.5 times larger, but possesses a 2.5 times larger eye than the *D. melanogaster* female. (Data are provided at doi.org/10.17617/3.1D).

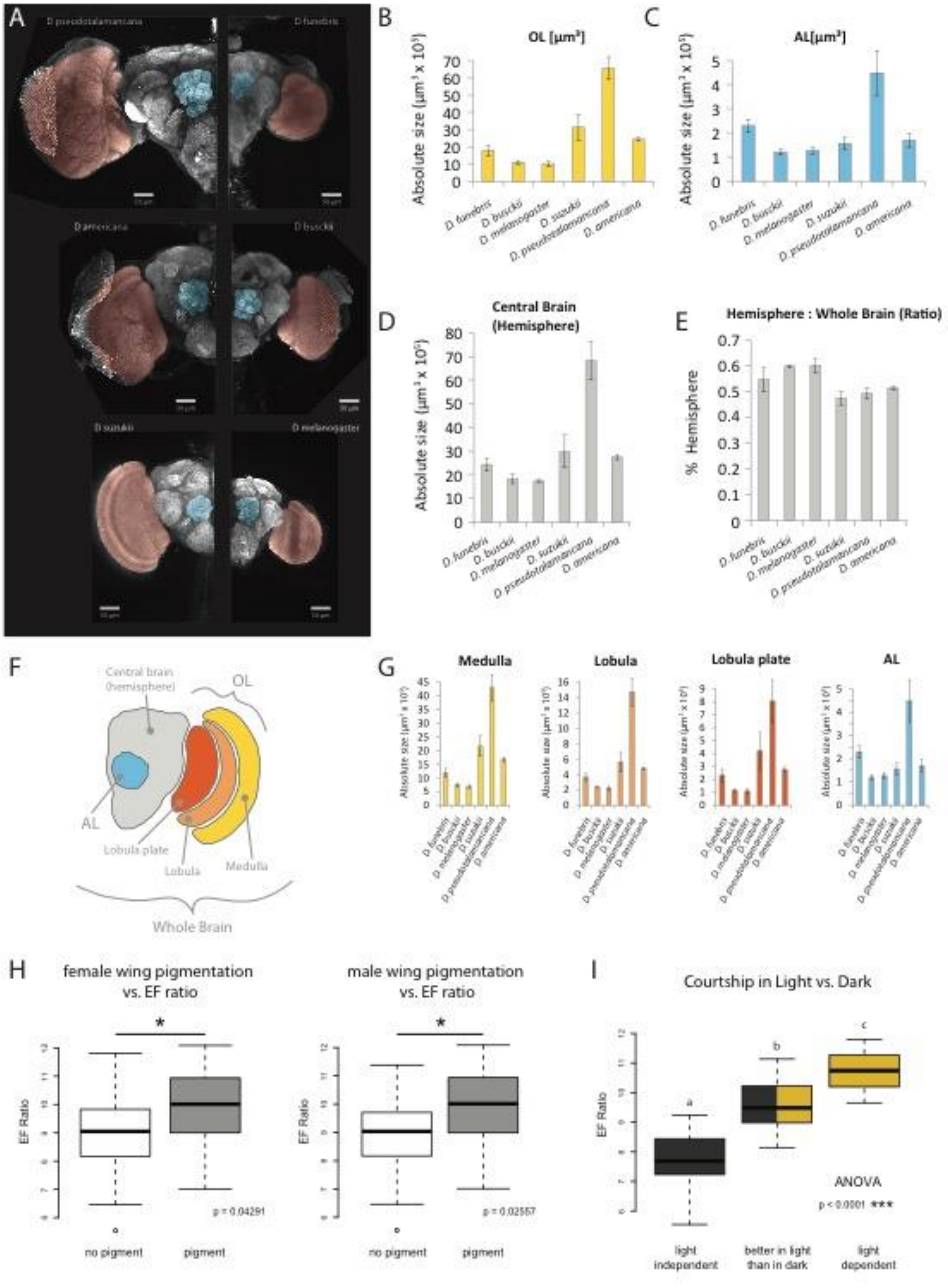

Supplementary Figure 3: Optic and antennal lobe measurements from 6 species. Red to yellow color signifies vision or visual bias, while blue indicates olfaction or olfactory species. (A) Confocal scans of each *Drosophila* species, with colored highlights for optic lobe (OL; red) and antennal lobe (AL; blue). Shown are the absolute measures of optic lobe (B), antennal lobe (C), and central brain volume (D), for each target species. (E) Although each species differed in absolute size, the ratio of central brain to total or whole brain (OL, AL, and central brain) for each species was roughly the same.

(F) Schematic of measurements taken from different species. (G) Absolute size of components of the OL and the AL from each species. (H) Female and male wing pigmentation plotted against EF ratio, where there is a correlation between relatively larger eyes and wing pigment across both sexes. An asterisk denotes statistical significance between two groups (*$P \leq 0.05$, ***$P \leq 0.001$; T-test). (I) Data from courtship in light or dark conditions as tested against EF ratio, where there is a highly significant difference in EF ratio across the three groups of courtship. Here again, relatively larger eyes correlate with better performance in light conditions, or with complete light-dependence for courtship. Means with the same letter are not significantly different from each other (ANOVA with Tukey-Kramer multiple comparison test). (Data are provided at doi.org/10.17617/3.1D).

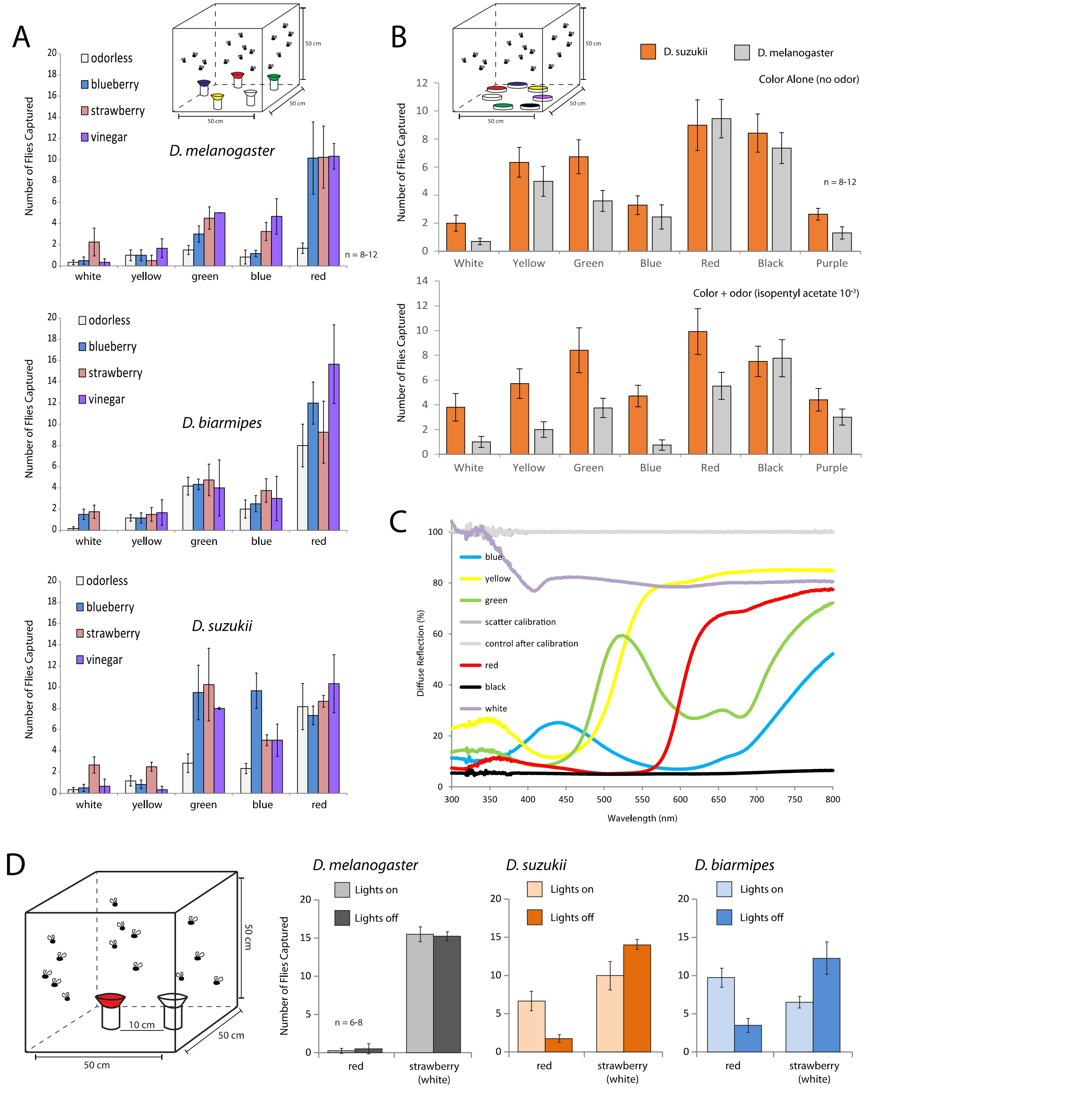

Supplementary Figure 4: Behavioral assays for visual and olfactory host navigation. (A) Design of trap assays using several visual and olfactory objects in testing attractive stimuli for each species. Red was the most attractive against the white background for all species regardless of the odor type, and even without odor, red was sufficient to capture spotted wing species. There was no significant difference in attraction to red when in combination with the three tested odors. The only color difference between species was noted to be an attraction to green for *D. suzukii*, as well as blue when in combination with blueberries, which they were reared upon. (B) Petri dish behavioral assay comparing *D. melanogaster* and *D. suzukii*, where both species showed similar color preference when presented without odor, although when with an odor, *D. suzukii* had a higher tendency towards white, yellow, green, blue and red than the other species. (C) Reflection index and wavelength for each color used in the behavioral assays. (D) Two-choice trap assay,

conducted in either full light, or full darkness. With lights off, all tested species were able to successfully navigate to the odor source; however, with lights on, the spotted wing species often mistakenly selected the visual object and not the odor object containing the fruit or food source, suggesting perhaps a visual bias or preference. In contrast, *D. melanogaster* always navigated to the odor source regardless of light condition or visual object, suggesting an olfactory bias or priority for this sensory cue. (Data are provided at doi.org/10.17617/3.1D).

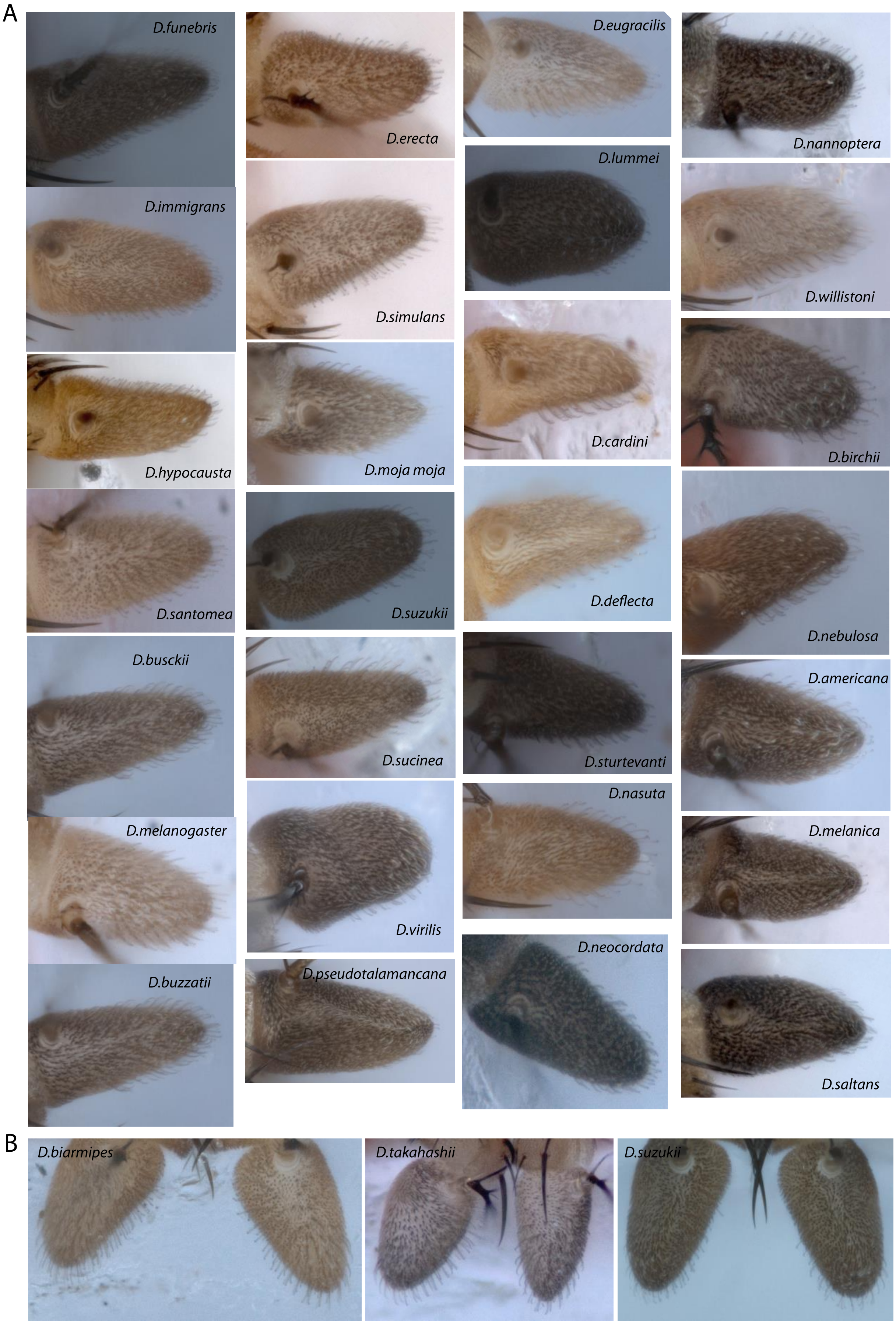
A
D.funebris
D.erecta
D.eugracilis
D.nannoptera
D.immigrans
D.simulans
D.lummei
D.willistoni
D.hypocausta
D.moja moja
D.cardini
D.birchii
D.santomea
D.suzukii
D.deflecta
D.nebulosa
D.busckii
D.sucinea
D.sturtevanti
D.americana
D.melanogaster
D.virilis
D.nasuta
D.melanica
D.buzzatii
D.pseudotalamancana
D.neocordata
D.saltans
B
D.biarmipes
D.takahashii
D.suzukii

Supplementary Figure 5: Antennal preparations and trichoid counts from selected species. (A) Each *Drosophila* species was mounted using single-sensillum recording (SSR) preparation techniques, and a series of images was taken to generate a z-stack photomontage. Trichoid sensilla were counted from male individuals over the same region of the funiculus for each *Drosophila* species. Images were taken with the arista mounted upward for consistency and for the best viewing angle as previously described for this sensillum type [2]. (B) Example of *Drosophila* species from a single phylogenetic clade that show a decreasing number of trichoid sensillum (left to right), and differences in surface area containing these sensilla, as well as differing sensillum length.

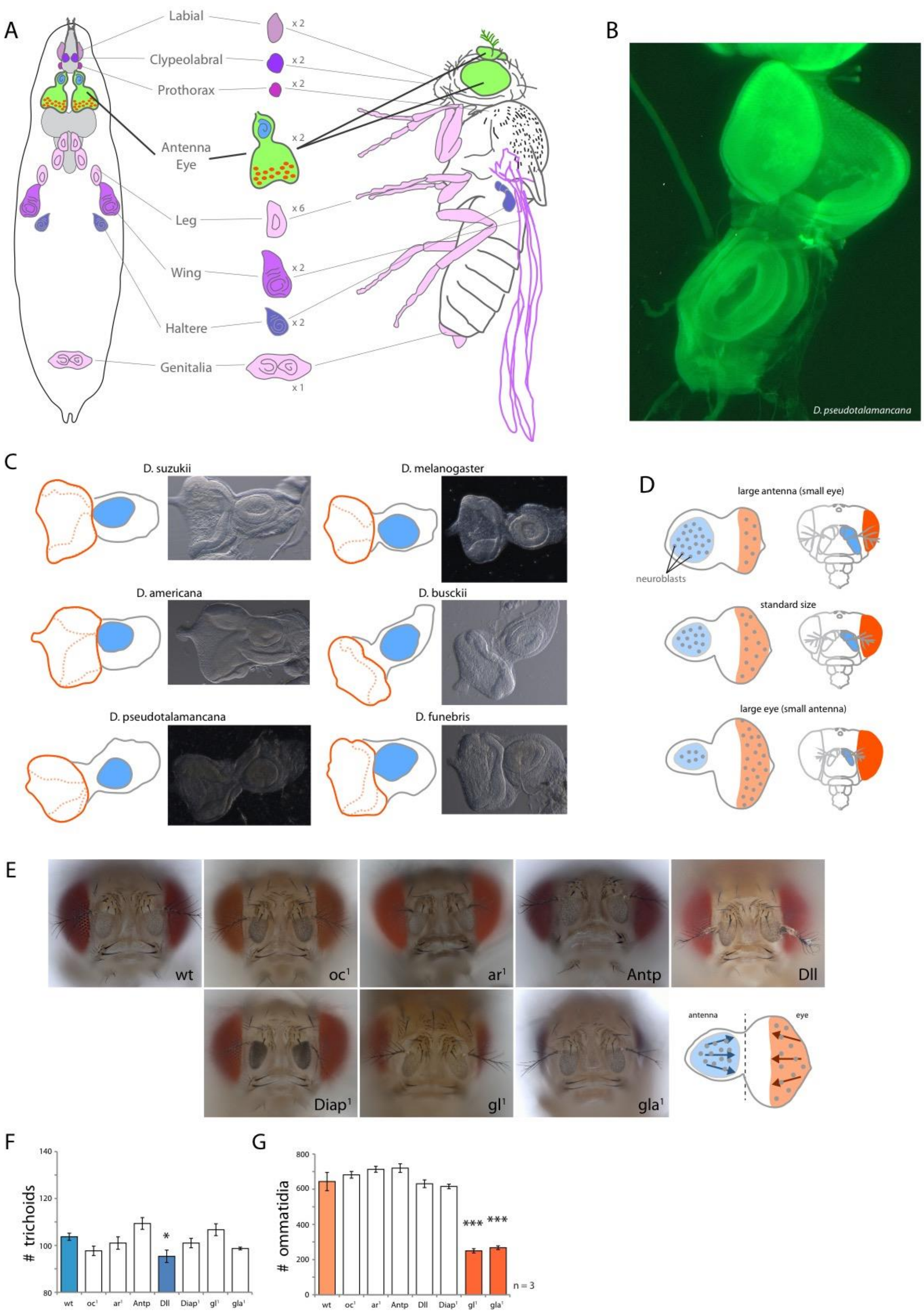

Supplementary Figure 6: The eye-antennal imaginal disc. (A) Diagram of the 19 total imaginal discs from *Drosophila* larvae and their corresponding location on the adult, highlighting that only one disc gives rise to two separate adult structures, namely the eye-antennal disc. (B) GFP labeling of *D. pseudotalamancana* imaginal disc, used to visualize the three-dimensional folding of the eye portion, as well as the shape and border of the antennal portion within the disc. (C)

Outlines and relative size measurements for eye and antenna from the imaginal discs of all 6 main species. Red color signifies vision or the visual system, while blue indicates olfaction. (D) Illustration of evo-devo theory of inverse resource allocation within one disc in order to generate a negative correlation between two adult sensory systems, the eye and antenna. (E) Wildtype and *melanogaster* mutants screened for either eye or antenna development, focusing on the ommatidium and trichoid numbers. (F) Trichoid number for each tested mutant, where only one was significantly different, Dll, which has an enlargement of the arista, and a decrease in each antennal segment size. Asterisk denotes significant difference from wildtype flies (T-test). (G) Ommatidium numbers from each mutant compared to the wildtype, where two lines showed marked reduction in ommatidia development. Asterisk denotes significant difference from wildtype flies (T-test). (Data are provided at doi.org/10.17617/3.1D).

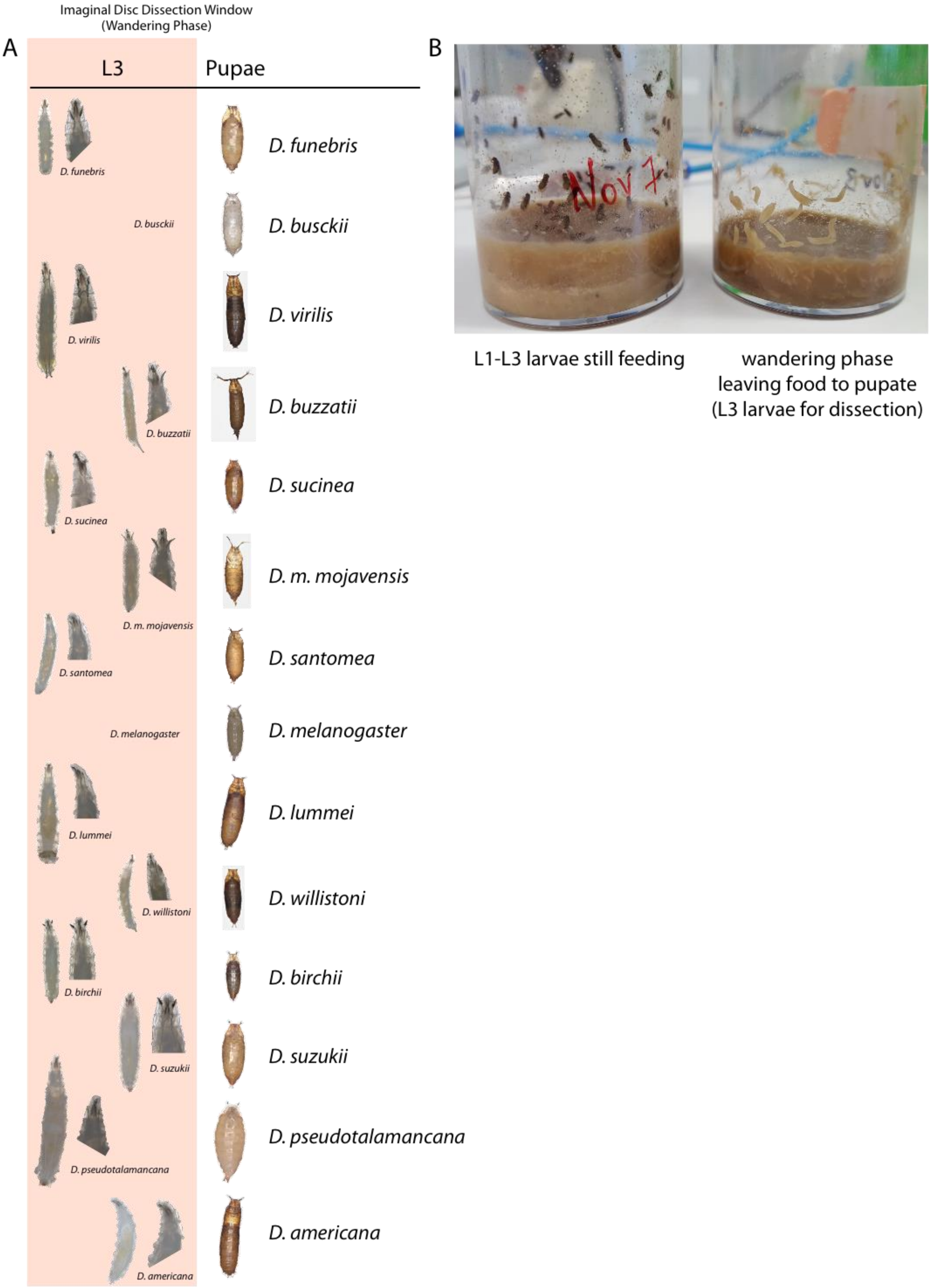

Supplementary Figure 7: Pupae and 3rd instar wandering phase larvae. (A) Given that each species had a different

developmental duration from egg to adult, we selected larvae for imaginal disc dissection during the same developmental window of time, namely the 3rd instar wandering phase larvae, which occurs just prior to the onset of pupation. (B) Example of 3rd instar larvae feeding on top layer of food (left) and 3rd instar wandering phase larvae (right) that have stopped feeding and are in search of a suitable pupation site. The latter of which were selected from each species for consistent dissection of the imaginal disc. (Data are provided at http://doi.org/10.17617/3.1D)

Supplementary Table 1: All scientific names, rearing media and stock numbers. (A) *Drosophila* species in alphabetical order, in conjunction with media used for rearing, as well as stock center identity. More information about each species is available through these stock numbers (e.g. site of insect collection, collection date, and reference specimens) (B-C) Recipe for diets used in this study. Green and blue colored diets were supplemented with either *Opuntia* cactus powder or fresh blueberries to enhance oviposition. Flies were maintained in a density-controlled manner, with 20-25 females per vial.

A

	Species Name	Diet/Media	UCSD/Cornell Stock #
1	*Drosophila affinis*	banana food	14012-0141.00
2	*Drosophila americana*	banana food	15010-0951.00
3	*Drosophila ananassae*	normal food	14024-0371.12
4	*Drosophila arizonae*	banana food	15081-1271.33
5	*Drosophila biarmipes*	normal food	14023-0361.10
6	*Drosophila birchii*	normal food	14028-0521.00
7	*Drosophila bromeliae*	banana food	15085-1682.00
8	*Drosophila busckii*	banana food	13000-0081.00
9	*Drosophila buzzatii*	normal food	15081-1291.02
10	*Drosophila cardini*	banana food	15181-2181.03
11	*Drosophila deflecta*	banana food	15130-2018.00
12	*Drosophila elegans*	normal food	14027-0461.00
13	*Drosophila erecta*	normal food	14021-0224.01
14	*Drosophila eugracilis*	normal food	14026-0451.02
15	*Drosophila ficusphila*	banana food	14025-0441.01
16	*Drosophila funebris*	normal food	15120-1911.05
17	*Drosophila gaucha*	banana food	15070-1231.03
18	*Drosophila hamatofila*	banana food	15081-1301.05
19	*Drosophila hydei*	normal food	15085-1641.03
20	*Drosophila hypocausta*	normal food	15115-1871.04
21	*Drosophila immigrans*	normal food	15111-1731.00
22	*Drosophila lummei*	wheat food	15010-1011.01
23	*Drosophila macrospina*	wheat food	15120-1931.00
24	*Drosophila mainlandi*	banana food	15081-1315.02
25	*Drosophila malerkotliana*	banana food	14024-0391.00
26	*Drosophila melanica*	normal food + blueberry	15030-1141.03
27	*Drosophila melanogaster Canton S*	normal food	Hansson Lab Strain
28	*Drosophila mercatorum*	normal food	15082-1521.00
29	*Drosophila mettleri*	banana food	15081-1502.11
30	*Drosophila mojavensis baja*	Banana-Opuntia	15081-1351.30
31	*Drosophila mojavensis mojavensis*	Banana-Opuntia	15081-1352.10
32	*Drosophila mojavensis sonorensis*	Banana-Opuntia	15081-1352.32
33	*Drosophila mojavensis wrigleyi*	Banana-Opuntia	15081-1352.30
34	*Drosophila montium*	banana food	14028-0701.00
35	*Drosophila mulleri*	Banana-Opuntia	15081-1371.01
36	*Drosophila nannoptera*	banana food	15090-1692.00
37	*Drosophila nasuta*	normal food	15112-1781.01
38	*Drosophila navojoa*	Banana-Opuntia	15081-1374.12
39	*Drosophila nebulosa*	normal food	14030-0761.00
40	*Drosophila neocordata*	banana food	14041-0831.00
41	*Drosophila pallidipennis*	banana food	15210-2331.01
42	*Drosophila polychaeta*	normal food	15100-1711.01
43	*Drosophila pseudoobscura*	banana food	14011-0121.00
44	*Drosophila pseudotalamancana*	normal food	15040-1191.00
45	*Drosophila putrida*	banana food	15150-2101.00
46	*Drosophila repleta*	banana food	15084-16611.02
47	*Drosophila repletoides*	banana food	15250-2451.01
48	*Drosophila robusta*	banana food	15020-1111.01
49	*Drosophila saltans*	banana food	14045-0911.00
50	*Drosophila santomea*	banana food	14021-0271.01
51	*Drosophila sechellia*	normal food + blueberry	14021-0248.07
52	*Drosophila simulans*	normal food	14021-0251.01
53	*Drosophila sturtevanti*	normal food	14043-0871.01
54	*Drosophila subobscura*	banana food	14011-0131.04
55	*Drosophila sucinea*	normal food	14030-0791.00
56	*Drosophila suzukii*	normal food + blueberry	14023-0311.01
57	*Drosophila takahashii*	normal food + blueberry	14022-0311.00
58	*Drosophila tsacasi*	banana food	14028-0701.00
59	*Drosophila virils*	normal food	15010-1051.00
60	*Drosophila wheeleri*	banana food	15081-1501.04
61	*Drosophila willistoni*	normal food	14030-0811.24
62	*Drosophila yakuba*	normal food	14021-0261.38

B

Normal Food

		500ml
treacle	g	59
brewer`s yeast	g	5.4
hot water	ml	101
agar	g	2.1
cold water	ml	135
Polenta	g	47
fill up with hot water	ml	135
flush out with hot water	ml	34
cold water	ml	54
propionic acid	ml	1.2
Nipagin 30%	ml	1.65

C

Banana Food

agar	g	85
yeast	g	165
methylparaben	g	13.4
blended bananas	g	825
Karo syrup	g	570
liquid malt extract	g	180
100% ethanol	ml	134
water	L	6

D

Wheat Food

		1L
semolina (corn based)	g	50
wheatgerm	g	50
sugar	g	50
dry yeast	g	40
agarose	g	8
water	ml	1000
propionic acid	ml	5
methylparaben	ml	3.3

Supplementary References

1. Sanchez-Gracia, A., Vieira, F. G., Almeida, F. C. & Rozas, J. in : *Encyclopedia of Life Sciences (ELS)* (2011). doi:10.1002/9780470015902.a0022848
2. Lin, C. & Potter, C. J. Re-classification of *Drosophila melanogaster* trichoid and intermediate sensilla using fluorescence-guided single sensillum recording. *PLoS One* **10** e0139675 (2015). doi:10.1371/journal.pone.0139675
