# Supplementary material for: Inverse resource allocation between vision and olfaction across the genus Drosophila
Source: Nat Commun. 2019 Mar 11;10:1162. doi: 10.1038/s41467-019-09087-z (PMC6411718; doi:10.1038/s41467-019-09087-z)
Supplement: Supplementary file 3 — Description of Additional Supplementary Files [file 41467_2019_9087_MOESM3_ESM.pdf]

Description of Additional Supplementary Files

File Name: Supplementary Data 1

Description: Drosophila species, genes and accession numbers for molecular phylogeny. Out of 16 nuclear and mitochondrial genes that were evaluated, we ultimately selected 5 genes to recreate the phylogeny for this genus. All gene evaluations are included with the online data library. Accession numbers for sequences are listed, as well as base pair (bp) length. Numbers in orange are partial sequences. Also listed after some accession numbers are the corrections made to the public annotations. Where available, sequences for all genes were pulled from the mitochondrial (mt) genome. (Source data are provided at http://doi.org/10.17617/3.1D)
