# Supplementary material for: Inverse resource allocation between vision and olfaction across the genus Drosophila
Source: Nat Commun. 2019 Mar 11;10:1162. doi: 10.1038/s41467-019-09087-z (PMC6411718; doi:10.1038/s41467-019-09087-z)
Supplement: Supplementary file 5 — Reporting Summary [file 41467_2019_9087_MOESM5_ESM.pdf]

nature research

Corresponding author(s): *Double-blind peer review submissions: write DBPR and your manuscript number here instead of author names.*

Last updated by author(s): *YYYY-MM-DD*

Reporting Summary

Nature Research wishes to improve the reproducibility of the work that we publish. This form provides structure for consistency and transparency in reporting. For further information on Nature Research policies, see Authors & Referees and the Editorial Policy Checklist.

Statistics

For all statistical analyses, confirm that the following items are present in the figure legend, table legend, main text, or Methods section.

n/a	Confirmed	
☐	☒	The exact sample size (*n*) for each experimental group/condition, given as a discrete number and unit of measurement
☒	☐	A statement on whether measurements were taken from distinct samples or whether the same sample was measured repeatedly
☐	☒	The statistical test(s) used AND whether they are one- or two-sided<br>*Only common tests should be described solely by name; describe more complex techniques in the Methods section.*
☒	☐	A description of all covariates tested
☐	☒	A description of any assumptions or corrections, such as tests of normality and adjustment for multiple comparisons
☐	☒	A full description of the statistical parameters including central tendency (e.g. means) or other basic estimates (e.g. regression coefficient) AND variation (e.g. standard deviation) or associated estimates of uncertainty (e.g. confidence intervals)
☒	☐	For null hypothesis testing, the test statistic (e.g. *F*, *t*, *r*) with confidence intervals, effect sizes, degrees of freedom and *P* value noted<br>*Give P values as exact values whenever suitable.*
☒	☐	For Bayesian analysis, information on the choice of priors and Markov chain Monte Carlo settings
☐	☒	For hierarchical and complex designs, identification of the appropriate level for tests and full reporting of outcomes
☒	☐	Estimates of effect sizes (e.g. Cohen's *d*, Pearson's *r*), indicating how they were calculated

Our web collection on statistics for biologists contains articles on many of the points above.

Software and code

Policy information about availability of computer code

Data collection	Does not apply
Data analysis	GraphPad InStat, and R

For manuscripts utilizing custom algorithms or software that are central to the research but not yet described in published literature, software must be made available to editors/reviewers. We strongly encourage code deposition in a community repository (e.g. GitHub). See the Nature Research guidelines for submitting code & software for further information.

Data

Policy information about availability of data

All manuscripts must include a data availability statement. This statement should provide the following information, where applicable:

- Accession codes, unique identifiers, or web links for publicly available datasets
- A list of figures that have associated raw data
- A description of any restrictions on data availability

Supplemental information including additional methodology, raw images and z-stack scans, molecular sequences, accession numbers, statistical assessments as well as species information are all available with the online version of the publication through the included DOI from the Max Planck Society hosted website: ("Edmond", the Open Access Data Repository of the Max Planck Society, http://doi.org/10.17617/3.1D)

Field-specific reporting

Please select the one below that is the best fit for your research. If you are not sure, read the appropriate sections before making your selection.

☐ Life sciences ☐ Behavioural & social sciences ☒ Ecological, evolutionary & environmental sciences

For a reference copy of the document with all sections, see nature.com/documents/nr-reporting-summary-flat.pdf

Ecological, evolutionary & environmental sciences study design

All studies must disclose on these points even when the disclosure is negative.

Study description	Comparison of 62 Drosophila species regarding their visual and olfactory system. The study includes the quantification of visual and olfactory structures (e.g. counting of ommatidia and olfactory sensilla) as well as the analysis of brain and developmental structures.
Research sample	The study includes the comparison of 62 Drosophila species that were selected to cover basically the full range along the phylogenetic tree of this genus.
Sampling strategy	Whenever it comes to the quantification of e.g. ommatidia or sensilla of numerous species, or the analysis of developmental stages we collected data of 8-10 samples per species. This number is in accordance with similar studies in the field. Qualitative analysis of brain structures were restricted to lower numbers due to the feasibility of a comparative study of so far unmatched 62 species from one genus.
Data collection	Different people were involved in the collection of data (e.g. the preparation of brains, antennae, eyes, etc.). Analysis was performed mainly by the first author. The obvious anatomical differences of some of the investigated fly species prevented us from performing double-blind data collection/analysis (which is common for anatomical studies).
Timing and spatial scale	All species are bred in the institute and data collection was performed within 1year prior to submission of the first manuscript.
Data exclusions	No data were excluded from the analysis.
Reproducibility	We did not repeat full experiments. However, e.g. the analysis of several brains of individual species revealed a very low intra-specific variability.
Randomization	To reduce the number of species for some more in-depth analyses, we used stratified random sampling to identify species that would represent all major subgroups of the genus.
Blinding	The obvious anatomical differences of some of the investigated fly species prevented us from performing double-blind data collection/analysis (which is common for anatomical studies).

Did the study involve field work? ☐ Yes ☒ No

Reporting for specific materials, systems and methods

We require information from authors about some types of materials, experimental systems and methods used in many studies. Here, indicate whether each material, system or method listed is relevant to your study. If you are not sure if a list item applies to your research, read the appropriate section before selecting a response.

Materials & experimental systems

n/a	Involved in the study	
☐	☒	Antibodies
☒	☐	Eukaryotic cell lines
☒	☐	Palaeontology
☐	☒	Animals and other organisms
☒	☐	Human research participants
☒	☐	Clinical data

Methods

n/a	Involved in the study	
☒	☐	ChIP-seq
☒	☐	Flow cytometry
☒	☐	MRI-based neuroimaging

Antibodies

Antibodies used	We used two staining antibodies called Hoechst & Phalloidin
Validation	Both antibodies are long-termed established antibodies for Drosophila.

Animals and other organisms

Policy information about studies involving animals; ARRIVE guidelines recommended for reporting animal research

Laboratory animals	We used 62 Drosophila species were all available commercially. All names are included in the manuscript. . Drosophila melanogaster mutants lines included: oc1 (ocelliless; Bloomington #2291), ar1 (arista-less; Bloomington #210), Antp (antennapedia; Bloomington #2235), Dll (distal-less; Bloomington #3306), Diap1 (thread; Bloomington #618), L1 (lobe; Bloomington #318), gl1 (glass; Bloomington #506), and gla1 (glazed; Bloomington #1951).
Wild animals	the study did not involve wild animals.
Field-collected samples	No field samples were included in this study.
Ethics oversight	No applicable for studies on Drosophila.

Note that full information on the approval of the study protocol must also be provided in the manuscript.
